# Left Atrial Appendage Anatomy: Implications for Endocardial Catheter-based Device Closure

**DOI:** 10.19102/icrm.2020.110704

**Published:** 2020-07-15

**Authors:** Abhishek Maan, E. Kevin Heist

**Affiliations:** ^1^Cardiac Arrhythmia Service, Massachusetts General Hospital, Boston, MA, USA

**Keywords:** Atrial fibrillation, left atrial appendage, stroke

## Abstract

This clinical review focuses on both current devices approved by the United States Food and Drug Administration and investigational devices available for left atrial appendage (LAA) closure. Specifically, the article describes the anatomical considerations that are particularly relevant from a procedural standpoint. In addition, we have also focused on the technical aspects of the procedure.

## Introduction

Stroke is one of the most common and debilitating complications in patients with atrial fibrillation (AF).^1^ Left atrial (LA) appendage (LAA) closure has emerged as an alternative to oral anticoagulation for thromboprophylaxis in patients with nonvalvular AF.^2^ Several technical and procedural considerations are important to achieve optimal procedural and postimplant success during LAA closure. Considering that the LAA is the major source of thrombus formation in the LA in patients with AF,^3,4^ the primary aim of LAA closure is to achieve complete disconnection between the LAA and systemic circulation to decrease the future risk of thromboembolism. The LAA anatomy is highly variable and precise knowledge of such is vital for accurate device selection and safe outcomes. Several anatomical factors such as shape, ostial diameter, and degree and distribution of septal trabeculations might affect both the immediate postprocedural and long-term follow-up outcomes after LAA closure. In addition, successful results following LAA closure are also reliant on knowledge of the anatomical relationship and proximity of the LAA with the neighboring structures. These factors can serve as a guide in preprocedural and intraprocedural planning to achieve successful outcomes. Preprocedural imaging can help with assessing the structural variations (eg, ostial diameter, depth, shape) in the LAA and interatrial septum [eg, presence of patent foramen ovale (PFO), atrial septal aneurysm], which can help to determine the feasibility of device closure along with the selection of type and size of the LAA closure device. In this review article, we discuss the implications of variations in the LAA anatomy from a procedural standpoint.

## Anatomical factors of the left atrial appendage

It is well-established that the morphology of LAA is highly variable. The overall complex anatomical structure of the LAA can be broadly divided into the trabecular and smooth regions, respectively. Most commonly, the LAA extends between the anterior and lateral walls of the LA, with its tip being directed anterosuperiorly and overlapping the left border of the right ventricular outflow tract.

The majority of LAAs have a well-defined orifice that leads to a neck region that opens to the body of the appendage. Although the usual shape of the orifice of the LAA is described to be oval, round, triangular, and water-drop shapes have also been reported, albeit less commonly.^5,6^

The different shapes of the LAA in a study based on cardiac magnetic resonance imaging (MRI) conducted in patients with chronic AF who underwent catheter ablation have also been previously described.^7^ In this study, a significant degree of interpatient variability in both the size and shape of the LAA was found.^7^ The findings were further supported by Wang etal. based on their observations using multidetector computed tomography (MDCT) and cardiac MRI. Based on the imaging characteristics, these authors classified the shapes of the LAA into four different categories: chicken wing, cactus, windsock, and cauliflower.^8^

Similarly, Koplay etal. classified the different shapes of LAA based on images of MDCT coronary angiography into seven different types: horseshoe (type 1), hand-finger (type 2a), fan (type 2b), wing (type 2c), hook (type 3), wedge (type 4), and swan (type 5).^9^ Another study incorporating postmortem heart specimens by Veinot etal. discussed the presence of lobes (which were adjudicated as protrusions from the main body); here, the presence of two lobes was described to be the most common finding (in 54%), followed by three lobes (23%), one lobe (20%), and four lobes (3%).^10^ In addition to the morphological variants, total LAA volume has been investigated as a risk factor of interest in patients with a history of stroke.^11^ A single-center study by Nedios etal. also described LAA based on its “takeoff” (superior versus inferior) in relation to its position in comparison with the left superior pulmonary vein.^12^ The heterogeneous nature of LAA morphology also underscores the fact that choosing closure devices for LAA based on a “one-size-fits-all” approach may not necessarily succeed in all cases.

## Left atrial appendage anatomy and the risk ofthromboembolism

The morphological variants of the LAA are believed to be implicated in the overall stroke risk for patients with either cryptogenic stroke or stroke without a clear predisposing cause. Variations in LAA morphology could potentially affect the blood flow velocities and subsequently influence the local coagulation milieu in the LAA.^13^ Taina etal. compared morphological variants of the LAA in patients with stroke. To mitigate AF as a potential confounding factor for stroke, only patients who were concluded to have a cryptogenic etiology or who were suspicious for a cardiac cause of such without any prior and/or current diagnosis of AF were included in this study. The investigators observed that the patients in the matched stroke subgroup had significantly higher LAA volumes (adjusted for body surface area, 5.7 ± 2.0mL/m^2^ versus 3.4 ± 1.1mL/m^2^; p < 0.001) when compared with the subgroup of patients without a history of stroke.^14^

**Figure 1:**
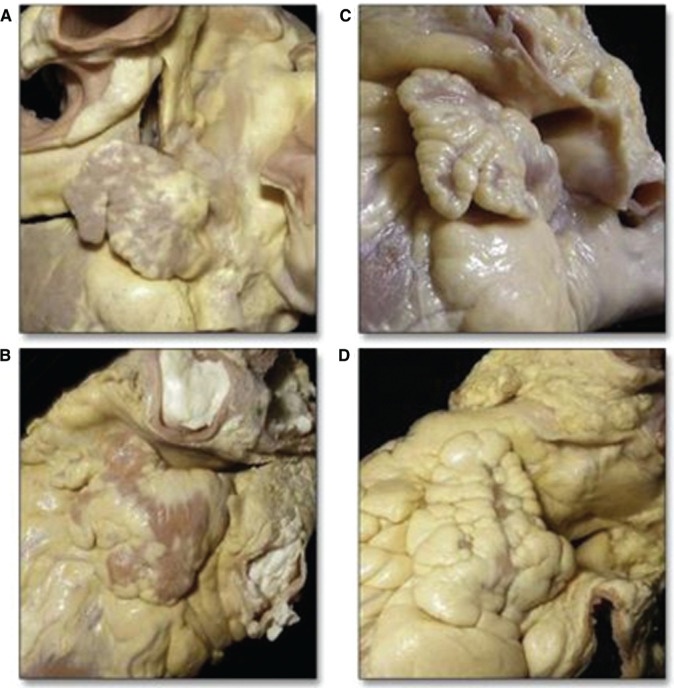
Variations in the anatomy of the LAA. **A:** Chicken-wing. **B:** Windsock. **C:** Cauliflower. **D:** Cactus. Reproduced with permission from Di Biase L, Santangeli P, Anselmino M, et al. Does the left atrial appendage morphology correlate with the risk of stroke in patients with atrial fibrillation? Results from a multicenter study. *J Am Coll Cardiol*. 2012;60(6):531–538.

These observations lend support to prior studies that obtained results based on computed tomography (CT) and MRI that also investigated the differential risk of thromboembolism according to morphological variants of the LAA. In a study involving 932 patients with AF, Di Biase etal. reported that the “chicken-wing” variant was associated with a 79% reduced likelihood of stroke/transient ischemic attack [odds ratio (OR): 0.21, 95% confidence interval (CI): 0.05–0.91; p = 0.036] after adjusting for CHADS_2_ score, gender, and type of AF. In another analysis, considering the “chicken-wing” variant as the reference category, the cactus morphology of the LAA was associated with a 4.08-fold greater risk (p=0.05), the windsock morphology was associated with a 4.5-fold greater risk (p = 0.04), and the cauliflower morphology was associated with an eightfold greater risk (p=0.05) of stroke/transient ischemic attack, respectively. In a subanalysis restricted to include only patients with CHADS_2_ scores of zero to one point(s) and adjusting for covariates such as gender, type of AF, and LA size, and a non–chicken-wing morphology was observed to be independent predictors for stroke (OR: 10.1, 95% CI: 1.25–79.7; p = 0.019).^15^ These findings could be of further relevance in patients found to have a lower risk for embolic phenomena using the CHADS_2_-VASc risk score. Various morphological variants of the LAA are shown in **Figure 1**.

## Left atrial appendage anatomy and endovascular closure

For closure with the WATCHMAN™ device (Boston Scientific, Natick, MA, USA), the diameter of the LAA ostium must be adequate enough to facilitate device compression by 8% to 20%. At some centers, the practice is to select the largest device permitted by the LAA depth to increase the likelihood of complete LAA occlusion while simultaneously avoiding excessively oversized devices (ie, ensuring device compression is not more than 30%–35% of the maximal ostial diameter). Usually, the size of the device for LAA closure is selected to be several millimeters wider than the largest measured LAA orifice diameter. A device–diameter mismatch typically requires resizing during the procedure and can potentially contribute to longer procedural times and an increased risk of complications. In a study based on cardiac MDCT data, Li etal. reported that there are significant variations in the shape of the LAA orifice. An oval shape was described to be the most common finding (in 81.5%), followed by triangular (7.3%); semicircular (4%); and, less commonly, round and foot-like shapes.^16^ In cases where a round device is implanted over an elliptical to ovoid-shaped LAA orifice, incomplete sealing of the orifice may potentially occur unless the device is sufficiently large enough to occlude the largest diameter of the LAA orifice.

The depth of the LAA is delineated as the distance from the midpoint of the line of the diameter to the most distal point in the LAA (ideally at the apex of the most anteriorly facing lobe). When the maximum depth of the LAA is shorter than the length of the smallest device that is wide enough to allow full closure, then LAA closure might not be technically feasible using the WATCHMAN™ device (Boston Scientific, Natick, MA, USA). Further, the distance between the orifice and the point at which the LAA initially deviates from its original course is also pertinent from the standpoint of ensuring effective device closure. Inadvertent deployment of the device too deep into the LAA can lead to hemopericardium and tamponade.^17^

## Association of the orifice and structure of the left atrial appendage with forms of atrial fibrillation

An autopsy-based study conducted by Ernst etal. showed that the LAA volume (as assessed by casts made from synthetic resin) was significantly higher (7,060mm^3^) in subjects with a prior history of AF (4,645mm^3^; p < 0.01).^6^ To an extent, these findings seem somewhat intuitive and could potentially be due to structural remodeling of the LAA in the setting of AF. It is also worthwhile to keep in mind that the LAA orifice area tends to be larger in patients with persistent forms of AF. Long-standing persistent AF additionally has an impact on the eccentricity of the LAA (in that persistent AF is associated with a progressive decrease in the eccentricity index in comparison with paroxysmal AF). In their study, Nucifora etal. observed that the persistent form of AF was associated with a larger orifice area (4.71± 0.94cm^2^ in permanent versus 3.44 ± 0.80cm^2^ in persistent versus 2.04 ± 0.48cm^2^ in paroxysmal AF, respectively).^5^ Similar findings in patients with persistent AF also having a larger orifice area (5.9 ± 2.8cm^2^ versus 4.4 ± 2.1cm^2^ in paroxysmal AF; p < 0.007) were observed in a study by Kishima etal. In addition, these investigators also noted that non–chicken-wing morphological variants were more common in patients with persistent AF than paroxysmal AF (39.5% versus 15.7%).^18^ From an implantation standpoint, the duration of AF and its correlation with the LAA orifice and shape could also be additional factors to consider.

## Preprocedural planning

If available, a three-dimensional CT or MRI scan can be used for anatomical assessment prior to the procedure, although such is not required for successful LAA closure. In a single-center study based on 53 patients, three-dimensional CT was observed to be superior to three-dimensional transesophageal echocardiography (TEE) for device size selection. In this study, making decisions based on the use of TEE alone would have led to the inappropriate exclusion of 10 of the 53 patients studied.^19^ Additionally, in the Prospective, Randomized Comparison of Three-dimensional CT Guidance Versus TEE Data for LAA Occlusion (PRO3DLAAO) trial, the use of CT was associated with greater accuracy in device selection on the first attempt (92% versus 27% with two-dimensional TEE; p = 0.01). Further, the overall procedural time was shorter with the use of three-dimensional CT (55 ± 17minutes versus 73 ± 24minutes with two-dimensional TEE; p = 0.05). The majority of the excess time in the two-dimensional TEE group of patients was spent on the additional deployment of device(s) and exchange of catheters.^20^

## Currently available devices and techniques

### WATCHMAN™

**Figure 2:**
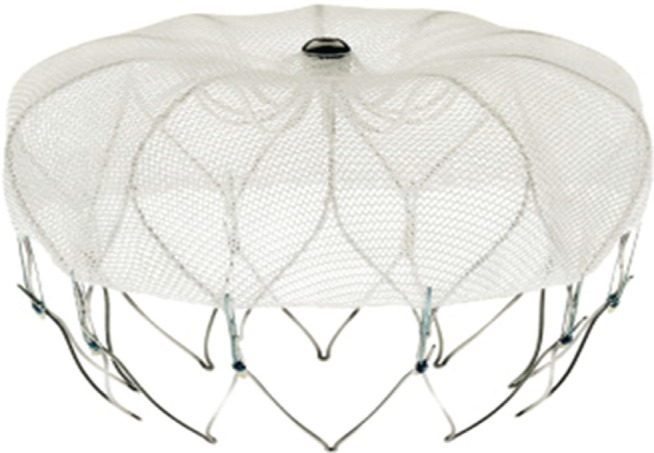
The WATCHMAN™ device (Boston Scientific, Natick, MA, USA).

The WATCHMAN™ device (Boston Scientific Inc., Natick, MA, USA) consists of a nitinol frame structure along with fixation barbs and a polyethylene terephthalate membrane that covers the surface facing the LA **(Figure 2).** Currently, the device is available in five different sizes ranging from 21mm to 33mm. Since the publication of the randomized controlled Watchman™ LAA System for Embolic Protection in Patients with AF (PROTECT-AF) trial, the WATCHMAN™ device remains the most frequently used option for LAA closure at this time. The rate of successful device implantation (with minimal peri-device leak < 5mm) in the PROTECT-AF trial was 88%.^21^

Based on a recent publication, the long-term (fie-year) follow-up data from both the PROTECT-AF and WATCHMAN™ LAA Closure Device in Patients with AF Versus Long-term Warfarin Therapy (PREVAIL) trials show that LAA closure with the WATCHMAN™ device was comparable to warfarin for the prevention of stroke. In addition, there were additional reductions in major bleeding, hemorrhagic stroke, and mortality.^22^ A second generation of the device is currently undergoing clinical investigation.

## Investigational devices

### WaveCrest®

**Figure 3:**
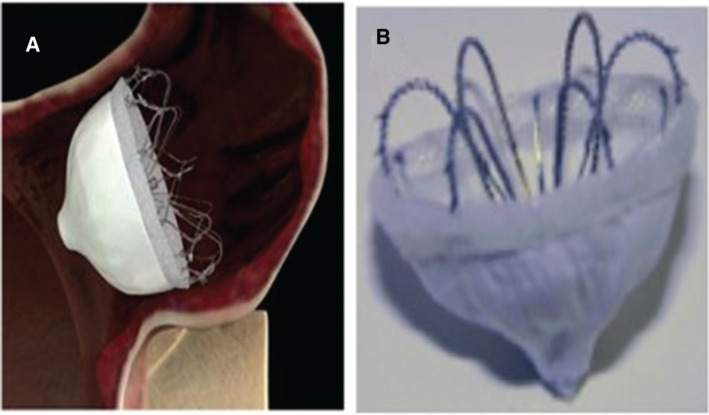
The WaveCrest™ device (Coherex Medical, Salt Lake City, UT, USA).

The WaveCrest® device (Coherex Medical, Salt Lake City, UT, USA) is shaped similar to an umbrella. It has a nitinol frame with an expanded polytetrafluoroethylene cover over a foam backing with exposed and flexible fixation anchors **(Figure 3)**. Currently, this device is available in three sizes (22, 27, and 32mm). In the Coherex WAVECREST I Left Atrial Appendage Occlusion Study, the initial experience with this device involving 73 patients with a successful implantation rate of 96% was reported.^23^ The WaveCrest® device is currently being investigated in a separate multicentric randomized clinical trial [WaveCrest® versus Watchman™ Transseptal LAA Closure to Reduce AF-mediated Stroke 2 (WAVECREST2)], with the WATCHMAN™ device (Boston Scientific Inc., Natick, MA, USA) in the control arm.^24^

### AMPLATZER™ Amulet™

**Figure 4:**
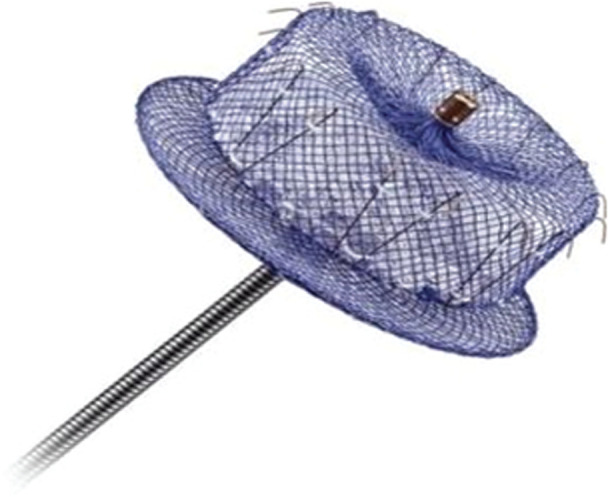
The AMPLATZER™ Amulet™ device (Abbott Laboratories, Chicago, IL, USA).

The AMPLATZER™ Amulet™ device (Abbott Laboratories, Chicago, IL, USA) is another endocardial device that has gained widespread use in Europe. The device has a proximal disc that is intended to achieve adequate closure of the LAA **(Figure 4)** and, as it is also described to require a depth of just 10mm for deployment, it may be preferable in patients with a shallow LAA landing zone.

The largest experience involving this device was a prospective study that had enrolled a total of 1,047 patients from 22 centers in Europe. The implantation success rate in this study was 97.3%, with a 4.97% incidence of periprocedural complications. In this study, no deaths were attributed to the device implantation.^25^ In a recently published multicentric, prospective study in 86 patients with contraindication to the use of warfarin, the implantation of the AMPLATZER™ Amulet™ device was safe and effective. In this study, with an average follow-up period of more than four years ± one year, seven patients (6.9%) experienced a major stroke (two cases per 100 patient-years versus an expected rate of 6.4 cases per 100 patient-years). There were no cases of late adverse events that could be directly attributed to device implantation.^26^ The AMPLATZER™ Amulet™ device is currently being investigated in the randomized controlled AMPLATZER™ Amulet™ LAA Occluder Trial, which seeks to compare this device with the WATCHMAN™ device (Boston Scientific, Natick, MA, USA) in patients randomized in a 1:1manner.^27^

### Ultraseal

**Figure 5:**
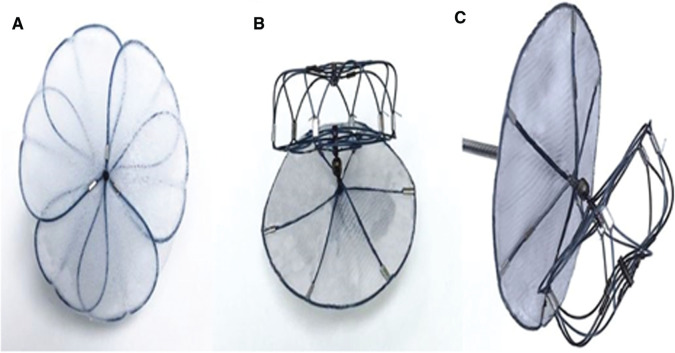
Ultraseal device (Cardia, Eagan, MN, USA).

The Ultraseal device (Cardia, Eagan, MN, USA) is a self-expandable device that consists of three different components (a distal bulb, a proximal sail, and an articulating center that connects the distal bulb and the proximal sail). An example of this device is presented in **Figure5**. Currently, the device is available in nine different sizes (16–32mm). From a deployment perspective, the manufacturer recommends that, ideally, the bulb diameter should be 25% to 33% larger than the intended landing zone to achieve optimal positioning.

There are accumulating data available regarding the implantation of the Ultraseal device. Regueiro etal. explored the feasibility of implanting this device in their single-center experience involving 12 patients with 45-day follow-up data, reporting no complications occurred except for device-related thrombus in one patient.^28^ Another smaller, single-center study by Pagnotta etal. also reported on the feasibility and safety of the Ultraseal device in their experience based on 23 patients with a mean follow-up of 166days ± 80days.^29^

### LAmbre™

**Figure 6:**
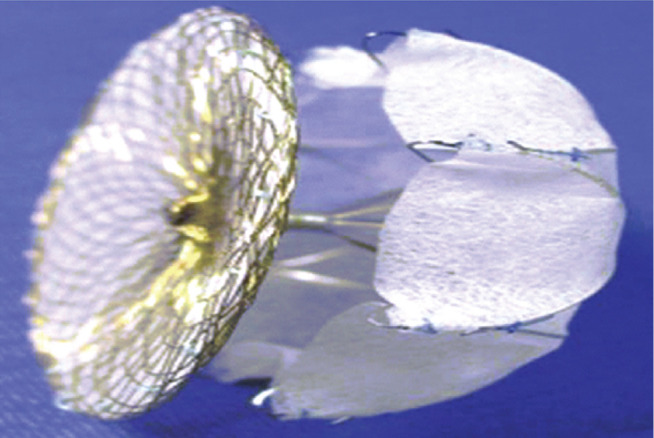
The LAmbre™ device (Lifetech Corp., Shenzen, China).

The LAmbre™ device (Lifetech Corp., Shenzen, China) is another novel self-expanding LAA occluder device composed of nitinol mesh and polyester membranes. Its structure is shaped similar to an umbrella and includes a cover connected by a short central waist. This device has a double stabilization design given the presence of eight small hooks that are distally located and another set of eight U-shaped ends that engage with the LAA trabeculations **(Figure 6)**. This device is available in 15 different diameter sizes ranging from 16mm to 36mm.

The LAmbre™ device was investigated in a prospective multicenter study with a reported implantation success rate of 99.35% (152/153 patients). During the one-year follow-up period of this study, ischemic stroke was observed in two patients and incomplete sealing of the LAA was noted in one patient, with no cases of device embolization.^30^ In another published prospective observational experience, LAmbre™ device recipients were compared with those who underwent AMPLATZER™ Amulet™ (n = 74) or WATCHMAN™ (n = 36) implantation. The investigators reported that patients in the LAmbre™ group (n = 30) had a higher incidence of complicated LAA morphology when compared with those in the other two groups (p = 0.006). From a procedural perspective, the overall success rate of implantation of this device was 99%, with a similar rate of device reposition during the procedure in comparison with the other two studied LAA closure devices.^31^

## Technical considerations during the procedure

Real-time imaging during the implantation of an LAA closure device remains critical for successful outcomes. TEE and intracardiac echocardiography can aid in successful site selection for the transseptal puncture and LAA deployment. Based on variations in the anatomy, the site of transseptal puncture varies from inferoposteriorly to mid- or superoposteriorly. To achieve adequate coaxial orientation, an inferoposterior approach might be a favorable strategy.

## Final device size selection

In the PROTECT-AF trial, adequate LAA closure was defined as any seal with a width of three 3mm or less (±2mm) of peridevice flow. In a post-hoc analysis of this trial, Reddy etal. investigated the incidence and clinical impact of incomplete LAA closure on peridevice residual blood flow. The comparison was performed to assess the impact of peridevice flow severity (minor < 1mm, moderate 1–3mm, major > 3mm) on a combined endpoint of stroke, systemic embolism, and cardiac mortality. During the follow-up assessment, adjudicating the patients with no peridevice leak as the reference group, there was no increased risk for the primary endpoint found among the patients with minor [hazard ratio (HR): 0.85, 95% CI: 0.11–6.40], moderate (HR: 0.83, 95% CI: 0.33–2.09), and major (HR: 0.48, 95% CI: 0.11–2.09) degrees of peridevice leak.^32^ From a practical standpoint, at our institution, we use information from intraprocedural TEE and contrast angiography of the LAA to assess the minimally acceptable device size. A significant oversizing of the LAA closure device compared to the ostium (> 30%–35% greater than the ostial diameter) could result in significant overdistension of the LAA and potential compression of surrounding structures. During implantation, the long axis of the LAA closure device is aligned with the long axis of the LAA and final device stability is confirmed using a tug test. After releasing the device from the delivery system, TEE imaging information is used to exclude possible complications [eg, device impingement on the mitral valve, compression of the left circumflex artery (LCx), pericardial effusion].

## Avoiding complications

### Coexisting patent foramen ovale

Currently, data on LAA closure in patients with coexisting PFOs remain limited. A retrospective experience by Gafoor etal. reported that closure of the LAA followed by closure of the PFO (11 patients), closure of both in the same setting (three patients), and closure of the PFO followed by closure of the LAA (three patients) are feasible.^33^ It is also worthwhile to note that, in the setting of coexisting PFO, it is likely that the trajectory of transseptal puncture might end up being superior and anterior. This is best avoided by incorporating imaging guidance.

### Anatomical considerations/proximity toneighboring structures

A thorough understanding of the anatomically proximal/neighboring structures of the LAA is crucial to avoid intraprocedural and postprocedural complications of LAA closure. One such anatomical relationship is the proximity of LAA to the LCx. Busquet etal. reported that, in approximately 30% of cases, LCx gives rise to the sinus node artery with close proximity to the LAA. In cases where these arteries course through the myocardium close to the ostium of the LAA, it is possible that these might be at a higher risk for peridevice trauma.^34^ Elsewhere, Li etal. also observed the close proximity of the LAA orifice and the proximal LCx (mean distance: 2.1 ± 0.9mm; range: 1.0–6.6mm).^14^ Although not reported in the context of LAA closure, vasospasm of the LCx has been suggested as a complication in 4% of cases in a study based on experience with cryoballoon ablation of the LAA in 100 consecutive patients.^35^

Another anatomically proximal structure that is of further relevance in regard to the LAA is the left phrenic nerve. In a study by Sanchez-Quintana etal., the left phrenic nerve was found to course along the pericardium, which overlies the LAA.^36^ Injury to the left phrenic nerve might be highest in patients undergoing epicardial closure of the LAA. Injury to the left phrenic nerve has been reported in the setting of catheter ablation, particularly during electrical isolation of the LAA.^37^ Considering that the LAA lacks a well-organized subendothelial layer, it hence has areas (anterior and anterolateral) that are relatively thin. These areas could potentially be more prone to perforation as a potential complication of LAA device closure.

## Conclusion

The field of LAA closure continues to rapidly evolve, with devices being introduced for use in patients with AF who are not suitable candidates for oral anticoagulation. Based on existing randomized controlled trials as well as multiple observational studies, the safety and feasibility of LAA closure devices continue to improve. It is crucial to have a thorough understanding of the LAA’s anatomy and its proximity to other cardiac structures to achieve optimal procedural outcomes and mitigate the risk of perioperative and postoperative complications.
